# Complete Genome Sequence of Helicobacter pylori Strain ATCC 43504, a Type Strain That Can Infect Gerbils

**DOI:** 10.1128/MRA.00105-20

**Published:** 2020-04-30

**Authors:** Ryo Kinoshita-Daitoku, Yoshitoshi Ogura, Kotaro Kiga, Fumito Maruyama, Tomoyo Kondo, Ichiro Nakagawa, Tetsuya Hayashi, Hitomi Mimuro

**Affiliations:** a Department of Infection Microbiology, Research Institute for Microbial Diseases, Osaka University, Osaka, Japan; b Division of Bacteriology, Department of Infectious Diseases Control, International Research Center for Infectious Diseases, The Institute of Medical Science, The University of Tokyo, Tokyo, Japan; c Department of Bacteriology, Graduate School of Medical Sciences, Kyushu University, Fukuoka, Japan; d Microbial Genomics and Ecology, Office of Academic Research and Industry-Government Collaboration, Academy of Hiroshima University, Hiroshima, Japan; e Department of Microbiology, Graduate School of Medicine, Kyoto University, Yoshida-Konoe, Sakyo, Kyoto, Japan; Portland State University

## Abstract

Helicobacter pylori ATCC 43504 is a type strain isolated from a gastric cancer patient in Australia and is commonly used for pathogenicity studies. In this study, we report the complete genome sequence of a strain that can infect gerbils. The data provide a basis for future H. pylori research.

## ANNOUNCEMENT

Helicobacter pylori is a Gram-negative spiral bacterium that inhabits the human gastric mucosa and is estimated to infect approximately half of the world’s population. H. pylori can persistently colonize the stomach, resulting in chronic gastritis, peptic ulcers, mucosa-associated lymphoid tissue (MALT) lymphoma, and gastric cancer (1). Here, we determined the genome sequence of ATCC 43504, which is a type strain of H. pylori. Though ATCC 43504 is also designated NCTC 11637 (2), these strains show different pathogenic properties, such as bacterial cell size, motility, adherence to cultured cells, and gastric infection efficiency (3). Therefore, it is important to determine the complete genome sequence of ATCC 43504 for not only understanding the virulence of H. pylori but also elucidating the functional differences of those type strains.

Strain ATCC 43504 was obtained from the American Type Culture Collection (Manassas, VA). H. pylori cells were cultured on 5% sheep blood agar (Trypticase soy agar with 5% sheep blood; BD, USA) under microaerobic conditions (5% O_2_, 15% CO_2_, and 80% N_2_) at 37°C for approximately 48 h. Colonies were collected and suspended in brucella broth (BD) containing 5% (vol/vol) inactivated fetal bovine serum (Thermo Fisher Scientific, USA). Cultures were agitated under microaerobic conditions at 37°C for approximately 15 h (4, 5). Genomic DNA was purified with the Genomic-tip 20/G kit (Qiagen, Canada) according to the manufacturer’s protocol.

The genome sequence of strain ATCC 43504 was determined using the 454 GS FLX Titanium system (Roche). A shotgun library and an 8-kb paired-end library were constructed using the Roche standard protocols and sequenced on a GS FLX+ instrument with Titanium chemistry. Low-quality sequences and adapters were removed using the GS Run Processor (Roche). A total of 189,473 single-end reads and 57,586 paired-end reads were assembled with GS Assembler software version 2.6 into a single scaffold containing 48 gaps. The gaps were closed, and the chromosome was circularized by sequencing gap-spanning PCR products using an ABI3130xl DNA sequencer (Applied Biosystems). To correct sequence errors, a paired-end library of the ATCC 43504 genome was constructed using the TruSeq DNA sample prep kit (Illumina) and sequenced with the Illumina MiSeq platform (2 × 150 bp). Low-quality sequences and adapters were removed using Platanus_trim (http://platanus.bio.titech.ac.jp/pltanus_trim). By mapping the obtained 221,588 reads to the initial genome sequence using BWA (6) and SAMtools (7), insertions/deletions were identified. To confirm and correct these candidate sequence errors, PCR was performed using primers designed to amplify each error candidate-containing region (Table 1), and the amplicons were purified by agarose gel electrophoresis and sequenced using an ABI3130xl DNA sequencer. Gene identification and annotation were conducted by the Microbial Genome Annotation Pipeline (MiGAP). Default parameters were used for all software.

**TABLE 1:** Primers used for PCR amplification for error correction

Primer	Sequence (5′–3′)	PCR product (bp)
ATCC_1f	TTGAATCCATGCCCGATATT	577
ATCC_1r	GCGTGGATGTAGAAGCCATT	
ATCC_2f	ATCAGTCTTGTGGCCAATCC	540
ATCC_2r	CCTTGGCAAAGTGCTTTAGG	
ATCC_3f	AGGGGCTTTTAGGTCATGCT	543
ATCC_3r	CTCAACATGGCATGGAATTG	
ATCC_4f	GCATTCCCACAAAGGCTAAA	567
ATCC_4r	ATCTCGCTCCAAACGCTAAA	
ATCC_5f	AGGCATAGGCCCAAGGTATT	543
ATCC_5r	TGTTCGCCCAACCTAAAAAG	
ATCC_6f	GCCTGAATTGCAAGCCTAAC	575
ATCC_6r	CCCTCCTATGGGGTTTCATT	
ATCC_7f	GCGATGCCAAAACTATCCAT	522
ATCC_7r	TCACATCAAGCATCCATCGT	
ATCC_8f	GCTTTTGCTCTTGGGTGTTT	507
ATCC_8r	CTCTTTGAGTGAGCGCATTG	
ATCC_9f	TAAAAGACGCCACCATAGGC	600
ATCC_9r	ATTCACATGCAAATCGCAAA	

The H. pylori ATCC 43504 assembly consists of a circular chromosome of 1,680,829 bp (GC content, 38.827%). The chromosome contains 1,615 coding sequences, 3 copies of rRNA genes (5S, 16S, and 23S), 36 tRNA coding regions, and 59 noncoding small RNAs. H. pylori ATCC 43504 contains a complete *cag* pathogenicity island (*cag*PAI) region carrying Western-type *cagA* (3,744 bp). The strain also encompasses *vacA* (3,873 bp), 2 copies of *babA* (2,049 bp and 2,235 bp), and *sabA* (1,908 bp). Although H. pylori strains are known to acquire genomic mutations easily, the genome size of ATCC 43504 is almost the same as that of NCTC 11637 (1,680,937 bp; GC content, 38.825%).

### Data availability.

The complete genome sequence of H. pylori strain ATCC 43504 has been deposited in GenBank under accession number AP017632. The raw data are available in the DDBJ Sequence Read Archive (DRA) under accession numbers DRA009796 and DRA009616.
